# Functional polymorphisms of xenobiotics metabolizing enzymes—a research topic

**DOI:** 10.3389/fgene.2013.00079

**Published:** 2013-05-08

**Authors:** José A. G. Agúndez, Kathrin Klein

**Affiliations:** ^1^Department of Pharmacology, University of ExtremaduraCáceres, Spain; ^2^Dr. Margarete Fischer-Bosch-Institute of Clinical Pharmacology, Stuttgart and Department of Clinical Pharmacology, University Hospital of TübingenTübingen, Germany

The human genome harbors an impressive number of genes encoding enzymes that primarily metabolize or transport drugs or other xenobiotics (XMEs). Genetic and functional variation in these genes is tremendous and has complex consequences, depending, for example, on whether enzyme structure or expression is affected, or whether the produced metabolite is pharmacologically or toxicologically active or not. Despite numerous impressive examples of the impact of genetic variation on pharmacokinetics and drug response, today's knowledge is incomplete regarding most XME genes and fragmentary even for many well-investigated XMEs. This is one of the reasons why clinical pharmacogenetic studies are often controversial and clinical application in personalized medicine is presently limited. Advanced technology and ongoing large-scale projects are rapidly uncovering the existing genetic variation in all populations on earth, ultimately enabling the personal genome in the very near future. A wealth of mostly rare novel variants is awaiting functional characterization either by high-throughput expression/phenotyping techniques or by prediction using improved algorithms to estimate functional relevance.

With this Research Topic we would like to give an up-to-date overview about the current knowledge in this field by covering both, known hard facts as well as cutting-edge advancement in novel genetic and genomic variation of XMEs and their functional consequences. Five major subtopics which include 20 research or review papers are included in this E-book. These are the following:

History and current knowledge of XMEs

- Clinical application of CYP2C19 pharmacogenetics toward more personalized medicine (Lee, 2013, review).
- Pharmacogenetics of cytochrome P450 2B6 (CYP2B6): advances on polymorphisms, mechanisms, and clinical relevance (Zanger and Klein, 2013, review).
- Pharmacogenetics of human ABC transporter ABCC11: new insights into apocrine gland growth and metabolite secretion (Ishikawa et al., 2013, review).
- Pharmacogenomics of cytochrome P450 3A4: recent progress toward the “missing heritability” problem (Klein and Zanger, 2013, review).

Clinical implications of XME gene variants

- *ABCB1* 4036A>G and 1236C>T polymorphisms affect plasma efavirenz levels in South African HIV/AIDS patients (Swart et al., 2012, research article).
- Genetic variations in drug-induced liver injury (DILI): resolving the puzzle (Stephens et al., 2012, opinion).
- MDMA, methamphetamine, and CYP2D6 pharmacogenetics: what is clinically relevant? (de la Torre et al., 2012, review).
- Molecular interactions between NAFLD and xenobiotic metabolism (Naik et al., 2013, review).
- Toward a clinical practice guide in pharmacogenomics testing for functional polymorphisms of drug-metabolizing enzymes. Gene/drug pairs and barriers perceived in Spain (Agúndez et al., 2012, perspective).

Inter/intraethnic variability of XME gene variants

- Characterization of the genetic variation present in *CYP3A4* in three South African populations (Drögemöller et al., 2013, research article).
- Frequencies of 23 functionally significant variant alleles related with metabolism of antineoplastic drugs in the Chilean population: comparison with Caucasian and Asian populations (Roco et al., 2012, research article).
- Pharmacogenomic diversity among Brazilians: influence of ancestry, self-reported color, and geographical origin (Suarez-Kurtz et al., 2012, review).

Regulation of XME gene expression

- Impact of the interaction between 3′-UTR SNPs and microRNA on the expression of human xenobiotic metabolism enzyme and transporter genes (Wei et al., 2012, research article).
- Molecular mechanisms of genetic variation and transcriptional regulation of *CYP2C19* (Helsby and Burns, 2012, review).

Pharmacogenetics in cancer therapy

- Impact of genetic polymorphisms on chemotherapy toxicity in childhood acute lymphoblastic leukemia (Gervasini and Vagace, 2012, review).
- Multilocus genotypes of relevance for drug metabolizing enzymes and therapy with thiopurines in patients with acute lymphoblastic leukemia (Stocco et al., 2013, review).
- Functional polymorphisms in xenobiotic metabolizing enzymes and their impact on the therapy of breast cancer (Vianna-Jorge et al., 2013, review).
- High-resolution melting analysis of the common c.1905+1G>A mutation causing dihydropyrimidine dehydrogenase deficiency and lethal 5-fluorouracil toxicity (Borràs et al., 2013, research article).
- Polymorphisms of phase I and phase II enzymes and breast cancer risk (Justenhoven, 2012, review).
- Analysis of the functional polymorphism in the cytochrome P450 *CYP2C8* gene rs11572080 with regard to colorectal cancer risk (Ladero et al., 2012, research article).
